# Supplementary material for: Mechanisms governing the pioneering and redistribution capabilities of the non-classical pioneer PU.1
Source: Nat Commun. 2020 Jan 21;11:402. doi: 10.1038/s41467-019-13960-2 (PMC6972792; doi:10.1038/s41467-019-13960-2)
Supplement: Supplementary file 7 — Source data [file 41467_2019_13960_MOESM7_ESM.zip › Source_Data/FigureS2/FigureS2B_MotifScanOutput/KmeansCluster7/homerResults/motif21.similar.html]

motif21

## Information for motif21

C
T
A
G
C
G
T
A
A
C
T
G
C
G
T
A
C
T
G
A
A
G
T
C
C
G
A
T
A
G
T
C
G
T
C
A
A
T
G
C
A
G
C
T
G
A
T
C
  
Reverse Opposite:  

C
T
A
G
C
T
G
A
T
A
C
G
A
C
G
T
A
C
T
G
C
G
T
A
A
C
T
G
A
G
C
T
G
C
A
T
T
G
A
C
C
G
A
T
G
A
T
C
  

|  |  |
| --- | --- |
| p-value: | 1e-55 |
| log p-value: | -1.274e+02 |
| Information Content per bp: | 1.759 |
| Number of Target Sequences with motif | 112.0 |
| Percentage of Target Sequences with motif | 3.49% |
| Number of Background Sequences with motif | 170.6 |
| Percentage of Background Sequences with motif | 0.39% |
| Average Position of motif in Targets | 81.7 +/- 52.0bp |
| Average Position of motif in Background | 102.9 +/- 72.2bp |
| Strand Bias (log2 ratio + to - strand density) | -1.4 |
| Multiplicity (# of sites on avg that occur together) | 1.02 |
| Motif File: | file (matrix) reverse opposite |

### Similar de novo motifs found

|  |  |  |  |  |  |  |  |
| --- | --- | --- | --- | --- | --- | --- | --- |
| Rank | Match Score | Redundant Motif | P-value | log P-value | % of Targets | % of Background | Motif file |
| 1 | 0.876 | C A T G C T G A A C T G G C T A C G T A T A G C C G A T T A G C G T C A T G A C G C A T | 1e-53 | -123.096346 | 4.58% | 0.80% | motif file (matrix) |
| 2 | 0.957 | G T C A A C T G C G T A C G T A A G T C A C G T A G T C C G T A A G T C A C G T | 1e-46 | -107.338677 | 2.87% | 0.31% | motif file (matrix) |
| 3 | 0.904 | A C T G C G T A C G T A A G T C A C G T A G T C C G T A A G T C A C G T | 1e-37 | -85.464119 | 2.65% | 0.36% | motif file (matrix) |
